# Gold Nanoparticles: An Efficient Antimicrobial Agent against Enteric Bacterial Human Pathogen

**DOI:** 10.3390/nano6040071

**Published:** 2016-04-14

**Authors:** Shahzadi Shamaila, Noshin Zafar, Saira Riaz, Rehana Sharif, Jawad Nazir, Shahzad Naseem

**Affiliations:** 1 Department of Physics, University of Engineering & Technology, Lahore 54890, Pakistan; noshin_zafer@yahoo.com (N.Z.); rsharif2002@yahoo.com (R.S.); 2 Centre for Excellence in Solid State Physics, University of Punjab, Lahore 54590, Pakistan; saira.cssp@pu.edu.pk (S.R.); shahzad_naseem@yahoo.com (S.N.); 3 Department of Microbiology, University of Veterinary and Animal Sciences, Lahore 54000, Pakistan; jawad.nazir@uvas.edu.pk

**Keywords:** gold nanoparticles, chemical reduction method, enteric bacterial human pathogens, gram-negative and gram-positive bacteria

## Abstract

Enteric bacterial human pathogens, *i.e.*, Escherichia coli, Staphylococcus aureus, Bacillus subtilis and Klebsiella pneumoniae, are the major cause of diarrheal infections in children and adults. Their structure badly affects the human immune system. It is important to explore new antibacterial agents instead of antibiotics for treatment. This project is an attempt to explain how gold nanoparticles affect these bacteria. We investigated the important role of the mean particle size, and the inhibition of a bacterium is dose-dependent. Ultra Violet (UV)-visible spectroscopy revealed the size of chemically synthesized gold nanoparticle as 6–40 nm. Atomic force microscopy (AFM) analysis confirmed the size and X-ray diffractometry (XRD) analysis determined the polycrystalline nature of gold nanoparticles. The present findings explained how gold nanoparticles lyse Gram-negative and Gram-positive bacteria.

## 1. Introduction

In recent years, nanoparticles (NPs) have become a promising candidate in place of conventional materials with enormous applications in the fields of science and engineering. The uniqueness of NPs is due to the higher surface-to-volume ratio and the increased number of atoms at their grain boundaries [1]. They proved to be significant materials in the advancement of various novel devices that are used in different biological, physical, pharmaceutical and biomedical applications [1,2,3]. The extraordinary functionality of NPs is mainly based on their size [4]. Among NPs, gold nanoparticles are widely used as a catalyst for medical therapy, gene therapy, and diagnostic and biological purposes [5,6,7]. The main advantage of gold NPs is that they are easy to synthesize by chemical reduction technique and they have a low toxicity compared to various nanomaterials. Different synthesis techniques have been adopted for various dimensions of nanoparticles and to functionalize their surface to improve their applications [4,8,9]. The main challenges in developing different strategies are their high purity and low polydispersity [4]. In order to control the size and shape of NPs, various reducing agents, stabilizers, and solvents, *etc.*, have been utilized in the preparation of NPs [10,11]. The control of particle size and morphology by adopting different stabilizers has been studied widely [12,13,14]. Gold NPs exhibit strong absorption spectra in the visible range, and this absorption is due to coherent oscillations of free electrons on the particle surface, which is called the surface plasmon resonance (SPR). The SPR spectra of gold NPs have enormous applications and have drawn great attention in recent years [15,16].

Recently, it has been investigated that the resistance of various enteric human pathogenic bacteria against many synthetic drugs is enhanced day by day [17]. Some reports on the antimicrobial activity of gold NPs against human bacterial pathogens have been published earlier, but the results are not significant for the zone of inhibition for E. coli and S. aureus which obtained only 7 mm and 16 mm, respectively [3,18]. Our attention is focused on evaluating the results against human pathogens of pure gold NPs with a small size; previously, no significant outcome with pure gold nanoparticles (GNPs) has been demonstrated. Hence, it is an attempt to investigate the efficiency of pure gold NPs with a precise dose against enteric bacteria, which has previously not been obtained.

Previously, the antibacterial activity of nickel nanoparticles against one type of bacterium (*E. coli*) has been investigated with the serial dilution method [19]. In the present project, nanometric-sized gold nanoparticles were synthesized by an easy and cost-effective chemical reduction process.

The main achievement of the current research is fabricating gold NPs of a small size by the reduction of NaBH_4_ instead of the familiar Turkevich method. Different doses of these NPs were utilized to check the antibacterial mechanism against four human pathogen bacteria: *E. coli*, *S. aureus*, *B. subtilis* and *Klebsiella pneumonia*. Then, from these doses, the zones of inhibition were studied by the Agar well diffusion method.

## 2. Experimental Section

### 2.1. Materials

All chemicals, chloroauric acid (HAuCl_4_·3H_2_O), sodium borohydride (NaBH_4_) and deionized water were purchased from Merk, India. All solutions were prepared in deionized water. The experiments for antibacterial activity were carried out using different bacterial strains. *Escherichia coli*, *Klebsiella pneumonia*, *Staphylococcus aureus*, and *Bacillus subtilis* were provided by the Microbiology Department, University of Veterinary and Animal Sciences, Lahore. Nutrient media was utilized for evaluating bacterial growth in liquid broth culture. A spectrophotometer was used to measure absorbance of bacterial growth at 600 nm.

### 2.2. Chemical Synthesis of Gold Nanoparticles

Gold nanoparticles were synthesized by employing sodium borohydride (NaBH_4_) as a reducing agent instead of old Turkevich or citrate method [20,21,22]. Two samples of gold nanoparticles (G1, G2) were fabricated by reduction of (0.2 mM, 40 mL) HAuCl_4_·3H_2_O with (0.050 M and 0.075 M, 0.02 mL) NaBH_4_. The solution of HAuCl_4_·3H_2_O (0.2 mM, 40 mL) was poured in a beaker and chilled solution of NaBH_4_ was added in it. Immediately the yellow color of gold salt solution converted into brick red color. Samples G1 and G2 were used as prepared. The chemical equation for the reaction is:

HAuCl_4_ + NaBH_4_ → Au + BH_4_ + HCl + NaCl_3_ (1)

The concentration of Au NPs is 67.72 μg/mL, which is calculated from Equation (1).

### 2.3. Antibacterial Activity

To observe the minimum inhibitory concentration (MIC) and minimum bactericidal concentration (MBC) of different-sized gold NPs, a simple procedure was followed. The effect of gold NPs on the kinetics of bacterial growth was examined by using enzyme-linked immunosorbent assay (ELISA) reader spectrophotometer. Nutrient Broth Dehydrated powder was used to make the nutrient broth (NB) medium. Sterility of all glassware and NB medium was performed by incubating for 24 h at 37 °C.

The transparent NB media (5.00 mL) was filled in the tubes and sterilized by autoclave for 15 min at 121 °C. The bacterial culture was prepared by shifting a known Gram-positive bacterial culture (*Bacillus subtillis* and *Staphylococcus aureus*), and Gram-negative bacteria (*Klebsiella pneumonia* and *Escherichia coli*) into different tubes containing NB media. The experiment incorporated a positive control (test tubes having gold NPs and nutrient broth media, without inoculum) and a negative control (test tube having inoculum and nutrient broth media, without gold NPs). The absorbance values for experimental test tubes (having nutrient broth media, inoculum and gold NPs) were corrected by deducting the corresponding absorbance values for the positive controls. All the experiments were performed in triplicate. The three doses were designed for the gold NPs solution. The 20 μL (low dose) solution contained 1.35 μg Au NPs, 30 μL (medium dose) contained 2.03 μg Au NPs and 40 μL (high dose) contained 2.70 μg Au NPs. Four tubes of each dose were prepared and incubated at 37 °C for 24 h.

A 200 μL from each tube was dispensed in each micro-well plate and observed by spectrophotometer. The optical density (OD) values from ELISA reader were taken in absorption mode which obscured the bacterial growth in each sample. Three values of OD for each sample and their mean were calculated with standard deviation.

For the calculation of zone of inhibition, a well diffusion method by Agar plates was used. Human pathogen bacteria *Escherichia coli* and *Staphylococcus aureus* were developed on nutrient Agar plate and maintained at 4 °C for the whole night. This overnight culture of bacteria in nutrient broth was then utilized in the experiment. In this technique, sterile nutrient Agar plate was equipped for each bacterium. These two bacterial pathogens were coated over the Agar plate with the help of sterile swab of cotton. Then these plates were permitted to dry. After that, four wells were bored by a sterile well cutter (6.0 mm dia.) in each agar plate. Subsequently, the suspension of NPs (5 μL, 15 μL and 30 μL) was poured into wells named 2, 3, and 4. The plates were allowed to place for 1 h for complete diffusion and then incubated at 37 °C for 24 h and measured the diameter of inhibitory zones in mm.

## 3. Characterization Techniques

UV-visible spectroscopy (UV-VIS, Spectro Dual split beam UVS 2700, Labomed Inc., Los Angeles, CA, USA) was employed for the optical analysis of gold nanoparticles. Atomic force microscopy (AFM, Veeco di Innova, Veeco instruments Inc., Plainview, NY, USA) analysis was performed in tapping mode under ambient conditions. X-ray diffractometry (XRD, PANalytical X’Pert Pro, Diffractometer, Almelo, the Netherland) analysis was used to confirm the structure. It was scanned in the range of 2θ (10°–80°).

## 4. Results and Discussion

### 4.1. UV-Visible Spectroscopy of Gold Nanoparticles

UV-visible absorption spectroscopy is the key toll to determine the structure and optical properties of metallic nanoparticles because the absorption bands associate the precise diameter and aspect ratio of metallic nanoparticles. Colloidal gold NPs have a distinctive red color solution. At nano-size, the surface electron cloud can vibrate and absorb the electromagnetic radiation of a particular energy. The samples of gold NPs were prepared by a chemical approach and the variation in the UV-visible spectrum of the resultant sol was observed to analyze the size effect of metallic nanoparticles on SPR.

**Figure 1:**
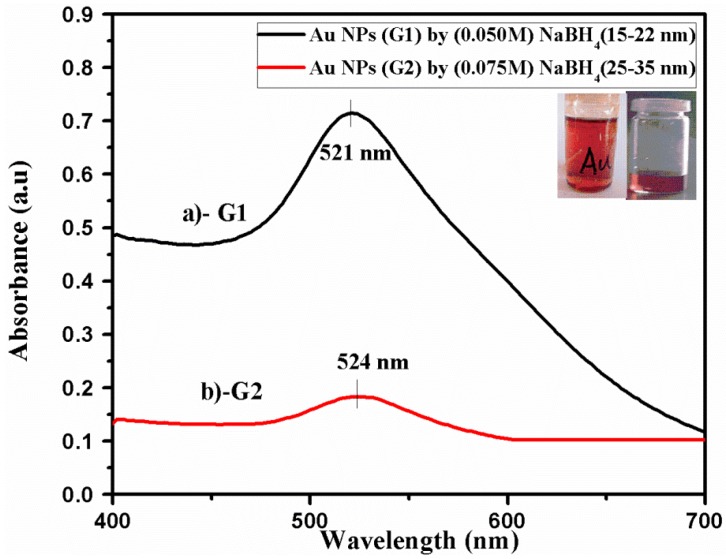
Ultra violet (UV)-visible spectrum of gold nanoparticles synthesized by the reduction of (**a**) 0.05 M NaBH_4_ (G1), (**b**) 0.075 M NaBH_4_ (G2) used as prepared.

Sample G1, a light pink solution, gives the SPR peak at the wavelength ~521 nm (Figure 1a). Sample G2, a bright red solution, gives the SPR peak at the wavelength ~524 nm (Figure 1b). The SPR peaks of the solutions of gold NPs with a diameter of ~15 nm exhibited the wavelength ~520 nm and gold NPs with a diameter of ~30 nm showed the absorbance peak at wavelength ~524 nm in the literature [23]. The shifting in SPR peak is congruent as the diameter of gold NPs increases. The SPR peak is damped for low concentrations of G2 NPs. The size range of both samples was confirmed by AFM analysis.

### 4.2. AFM Analysis of Gold Nanoparticles

**Figure 2:**
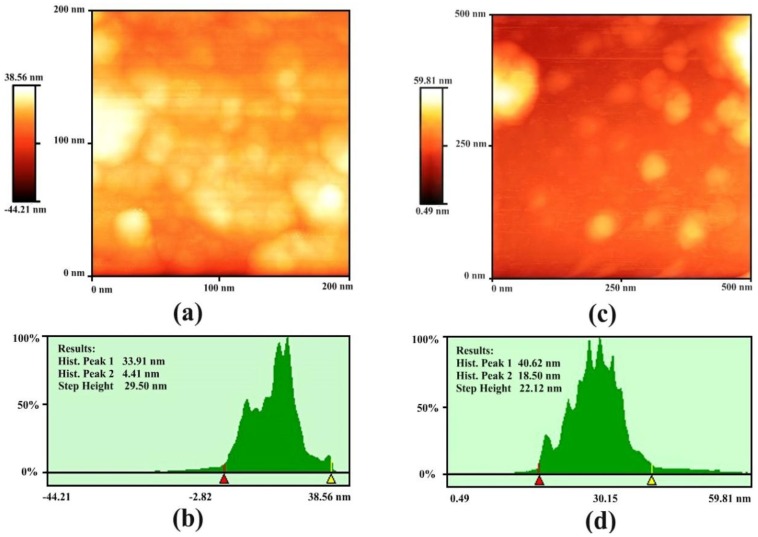
Atomic force microscopy (AFM) analysis of gold nanoparticles: (**a**,**b**) 2D image and histogram analysis of sample G1, respectively; (**c**,**d**) 2D image and histogram analysis of sample G2, respectively.

Atomic force microscopy (AFM) was executed for gold samples G1 and G2 to observe the presence of particles, particle size, and their size distribution. AFM analysis was performed in tapping mode and two-dimensional (2-D) image of nanoparticles is taken with a scan area of 0.5 × 0.5 μm as shown in Figure 2. The size of fabricated gold NPs (G1) ranges from 6 to 34 nm as observed by the analysis of the histogram (Figure 2b). Maximum particle ranges are found in the size range of 15–22 nm. This is congruent to the result of G1 (Figure 1a) obtained by UV-visible spectroscopy.

The sizes of the gold NPs of sample G2 have a range between 20 and 40 nm as observed by the histogram analysis of sample G1 (Figure 2c,d). Here, maximum particles are found in the size range of 25–35 nm, satisfying the result of UV-visible spectroscopy of G2 (Figure 1b).

### 4.3. XRD Analysis of Gold Nanoparticles

**Figure 3:**
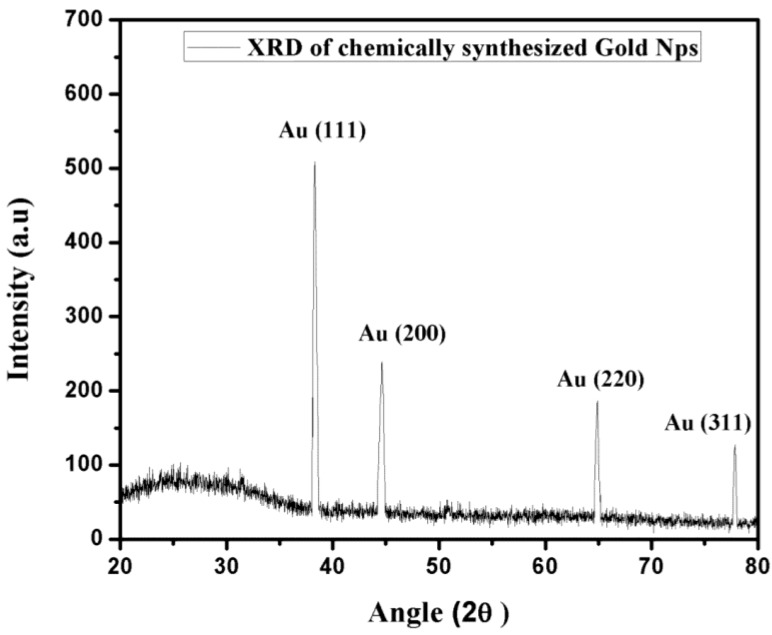
X-ray diffraction pattern of chemically synthesized gold nanoparticles.

The crystal structure of gold NPs is demonstrated by XRD as shown in Figure 3. Samples are prepared by the drop-casting of gold sol on a glass surface. The diffraction peaks of gold NPs are observed at 38.15°, 45.6°, 64.6° and 77.8°, representing the index as (111), (200), (220) and (311), respectively, which verified the polycrystalline face-centered cubic structure, and thus matched the literature [24].

### 4.4. Antimicrobial Mechanism of Gold Nanoparticles

The *in vitro* antibacterial mechanism of samples G1 and G2 was demonstrated by using the turbidity of the nutrient broth. The zone of inhibition was examined by taking optical density values (OD) from the spectrophotometer with a 600 nm filter. The three doses were employed: the low dose of 1.35 μg, the medium dose of 2.03 μg and the high dose of 2.70 μg gold NPs.

The mean OD values of all 48 test tubes of each sample were examined carefully. The effect of the antimicrobial mechanism of gold NPs on *S. aureus*, *K. pneumonia*, *E. coli* and *B. subtilis* was statistically calculated. The percentage (%) decrease or increase in the growth of the bacteria was measured against the OD value of pure (nutrient broth) media (reference OD value).

The percentage growth for the negative control (nutrient broth and inoculum) for *S. aureus*, *E. coli*, *B. subtillis* and *K. pneumonia*, after 24 h of incubation at 37 °C was 75.2%, 74.3%, 68.5% and 71.9%, respectively. In triplicate tubes for each low, medium and high dose with inoculum after 24 h incubation, the bacterial growth decreases with the increasing dose of nanoparticles (Table 1.).

**Figure 4:**
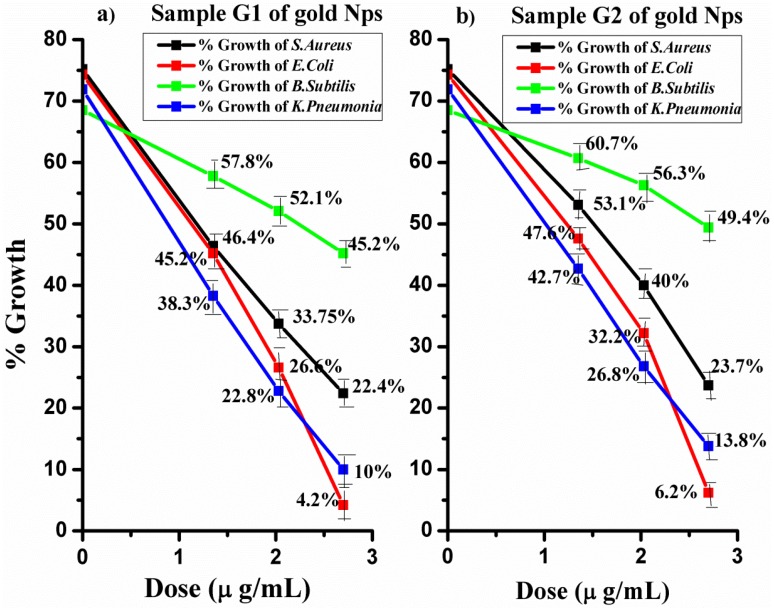
(**a**) Percent growth of *S. aureus*, *B. subtilis*, *E. coli*, and *K. pneumonia* by G1 of gold NPs; (**b**) Percent growth of *S. aureus*, *B. subtilis*, *E. coli* and *K. pneumonia* by G2 of gold NPs.

G1 gold NPs which had a smaller size (6–34 nm) than G2 NPs revealed the following reduction in growth. The maximum growth of *S. aureus* without any dose was 75.19%, which was reduced up to 22.4%, whereas *E. coli* with a maximum growth of 74.3% was reduced up to 6.2%. Thus, for complete inhibition of *S. aureus,* a larger dose is required. The reduction in growth of *B. subtilis* was only 45.2% and that of *K. pneuomonia* was reduced up to 10%. On the other hand, the reduction in growth by G2 with gold NPs with a size of 20–40 nm on *S. aureus* was up to 23.7% whereas *E. coli* was reduced up to 6.2%. The reduction in growth of *B. subtilis* was 49.4% and the growth of *K. pneuomonia* was reduced up to 13.8%, as exhibited in Figure 4.

The antibacterial effect of the two samples of gold NPs of different sizes was examined. The same doses of both samples of NPs provided maximum inhibition against each pathogen. G2 NPs are proved to be less competent as compared to G1 NPs, which may be due to the difference in size, thus reducing the antibacterial mechanism of G2.

The antimicrobial action of gold NPs for Gram-positive and Gram-negative bacteria is different. The main difference is the structure of the membrane *i.e.*, the peptidoglycan layer’s thickness, which is an important part of pathogenic bacteria. Its thickness is 50% higher in Gram-positive than in Gram-negative bacteria [25]. Thus, larger doses of nanoparticles are required for Gram-positive bacteria.

Gold NPs exerted the antibacterial effect in two steps. First, they changed the membrane potential and reduced adenosine triphosphate (ATP) synthase activities, thus reducing the metabolism process. Secondly, they declined the subunit of the ribosome for tRNA binding, thus collapsing its biological mechanism. At the same time, they proved to be less toxic to mammal cells [26]. Gold NPs with a small size and enhanced surface area produce some electronic effects which are beneficial for enhancing the surface reactivity of NPs. In addition, the high surface area percentage directly interacted with the microorganism to an enormous extent and hence provided an improved contact with the bacteria. These two important factors greatly enhanced the antimicrobial activity of the NPs with large surface area. Bacterial proteins of the cell wall and cytoplasm were responsible for the function of the cell. These NPs disturbed the normal functioning of these proteins to cause cell death. Gold NPs basically reacted with sulfur- or phosphorus-containing soft bases. Therefore, the sulfur-containing proteins and phosphorus-containing DNA molecules are favored sites for GNPs to attack. The gold NPs binded to thiol groups of enzymes such as nicotinamide adenine denucleotide (NADH) dehydrogenases and disrupted their respiratory chains with the release of oxygen species, producing oxidative stress. As a result, significant damage occurred in the cell structures and finally led to cell death [26].

The other important factors are the concentration and size of the nanoparticles, which play a vital role in the antibacterial mechanism. The antibacterial mechanism of gold NPs on gram-positive and gram-negative bacteria depends on the concentration of the gold NPs [27]. It was demonstrated that the catalytic and antibacterial mechanism of the gold NPs increases with a decrease in average size [21].

The current research revealed that the small-sized sample G1 of gold NPs exhibits maximum bacterial vulnerability compared to sample G2. This result is due to their small size, increased surface area and good penetrating power. They exhibit well-defined surface chemistry, high chemical stability and a well-defined smaller size, which make it easier for them to attach to the microorganisms. Specifically, the small-size NPs can easily bind to the important features of the outer membrane, thus producing changes in structure, degradation and, lastly, cell death.

Different doses of gold NPs exhibited higher antibacterial activity against *E. coli* as compared to other examined bacteria. *S. aureus* bacteria are more resistive as compared to *E. coli* and *K. pneumonia*, so a dose of 2.70 μg/mL is enough for the maximum inhibition of *E. coli* and *K. pneumonia.* Larger doses of gold NPs are required for the complete inhibition of *S. aureus* and *B. subtilis* due to their thick peptidoglycan layer. *B. subtilis* has been proved to be the most resistant Gram-positive bacteria because it has the ability to survive in any harsh conditions.

### 4.5. Statistical Analysis

The antibacterial effect of each dose of gold NPs was calculated statistically. The regression line and estimated value with standard error were also considered. All the experimentation was performed thrice. However, this experimental data was further investigated by applying the Anova test (*p* < 0.05).

### 4.6. Regression Line

The regression line was drawn by plotting doses of gold NPs along the *X*-axis and the % growth along the *Y*-axis. The regression line was examined by putting Table 1 values in Equations (2)–(4).

(2) $$a=\frac{\sum Y}{N}-b\frac{\sum X}{N}$$

(3) $$b=\frac{\sum XY-\frac{\sum X\sum Y}{N}}{\sum X^{2}-\frac{{\left(\sum X\right)}^{2}}{N}}$$

*Y* = a + b*X* (4)

From the above equations, growth inhibition curves were obtained for both samples of gold NPs.

**Figure 5:**
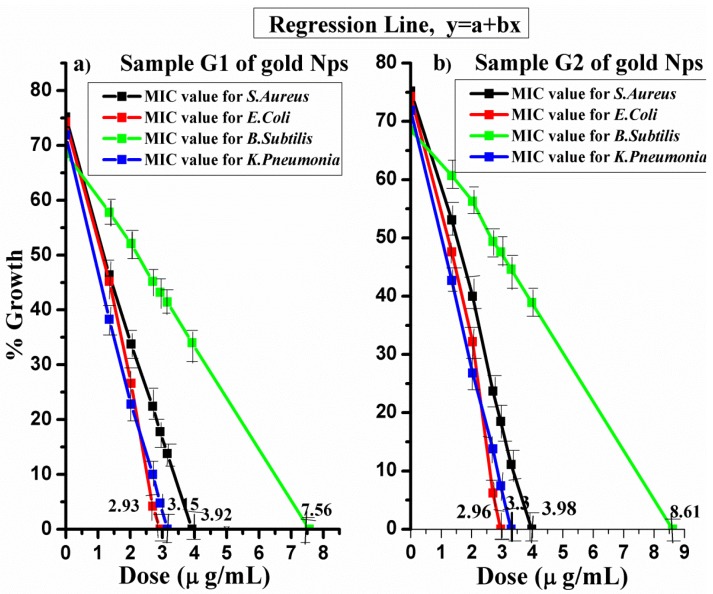
(**a**) Maximum inhibition concentration of sample G1 for *S. aureus*, *E. coli*, *B. subtilis* and *K. pneumonia*; (**b**) maximum inhibition concentration of sample G2 for *S. aureus* and *E. coli*, *B. subtilis* and *K. pneumonia* according to regression line.

From Figure 5, the minimum inhibitory concentrations (MICs) of sample G1 with sizes of 7–34 nm are 2.93 μg/mL, 7.56 μg/mL, 3.92 μg/mL, and 3.15 μg/mL for *E. coli*, *B. subtilis*, *S. aureus*, and *K. pneumonia*, respectively. The MICs of sample G2 with sizes of 20–40 nm are of 2.96 μg/mL, 8.61 μg/mL, 3.98 μg/mL and 3.3 μg/mL for *E. coli*, *B. subtilis*, *S. aureus*, and *K. pneumonia*, respectively.

The antibacterial mechanism of gold NPs with a size range of 40–80 nm using the Agar method of diffusion was observed [27] and the zones of inhibition of *E. coli* and *K. pneumonia* were found. *S. aureus* had a slightly smaller zone of inhibition as is demonstrated in current research.

These above doses were further utilized to calculate the zones of inhibition of *S. aureus* and *E. coli.*

**Figure 6:**
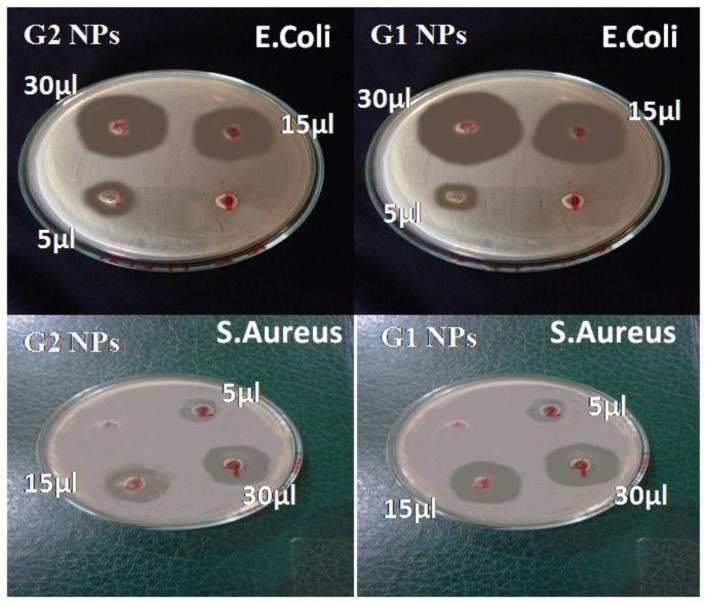
Well diffusion method of two sizes of gold nanoparticles (GNPs) against *E. coli* and *S. aureus.*

The well diffusion technique results of G1 NPs and G2 NPs against two bacteria, *E. coli* and *S. aureus*, are represented in Figure 6 and expressed in Table 2, which clearly demonstrate the inhibition zones for gold NPs that are missing in the control reading. Furthermore, the inhibition zone of G1 NPs is slightly larger than that of G2 NPs, as indicated in Table 2.

No significant result has been obtained from gold NPs in the literature as compared to silver NPs [28,29]. Selvaraj and his coworkers found 27 mm for the zone of inhibition of *E. coli* with citrate-capped gold NPs [30], while Nazari *et al.* calculated 14 mm and 13 mm for the inhibition zones for *E. coli* and *S. aureus* with 4000 μg doses [31]. The current research revealed that the zone of inhibition for gold NPs (G1NPs) sized 7–34 nm against *E. coli* and *S. aureus* is found to be ~31 mm and 22 mm, and for gold NPs sized 20–40 nm it is found to be ~35 mm and 25 mm, respectively. In a recent project, gold NPs exhibited excellent results and showed a maximum zone of inhibition against enteric bacterial pathogens with precise dose calculations.

## 5. Conclusions

In the current project, gold nanoparticles 6–40 nm in size exhibited high antibacterial activity compared to those reported earlier [3,17,18,19,27]. The mechanism of this activity was found to be size- and dose-dependent. It was more influential against Gram-negative bacteria. The antibacterial mechanism of gold NPs against four pathogenic bacteria demonstrated that gold NPs can be the next therapy against this enteric bacterium. The MICs (minimum inhibitory concentrations) of gold NPs 7–34 nm in size are 2.93 μg/mL, 7.56 μg/mL, 3.92 μg/mL, and 3.15 μg/mL for *E. coli*, *B. subtilis*, *S. aureus*, and *K. pneumonia*, respectively, whereas the MIC values of gold NPs 20–40 nm in size are 2.96 μg/mL, 8.61 μg/mL, 3.98 μg/mL and 3.3 μg/mL for *E. coli*, *B. subtilis*, *S. aureus*, and *K. pneumonia*, respectively. Similarly, the maximum zone of inhibition for gold NPs (GNPs) 7–34 nm in size against *E. coli* and *S. aureus* is 31 mm and 22 mm, and for gold NPs 20–40 nm in it is 35 mm and 25 mm, respectively. These nanoparticles enhance their potential in the fields of physics, biomedicine and chemistry due to their excellent, functionally active characteristics. In biomedicine, gold nanoparticles have been proved to be a vital revolution for drug delivery and cancer therapy. They also perform as a safe and non-toxic antimicrobial agent due to their functional nature as compared to antibiotics. Turbidity proves to be an efficient technique for the estimation of bacterial growth inhibition in a liquid. These MIC calculations are also supportive for calculating the maximum inhibition zone using the Agar disk diffusion method.

## Figures and Tables

**Table 1:** Antibacterial effect on *Staphylococcus* *aureus* (*S. aureus*), *Escherichia* *coli* (*E. coli*), *Klebsiella* *pneumonia* (*K. pneumonia*) and *Bacillus subtilis* (*B. subtilis*) for low, medium and high doses of gold nanoparticles (NPs) of two sizes, sample named as G1 and G2.

Strain	(%) Reduction in Growth Sample G1 of Gold NPs			(%) Reduction in Growth Sample G2 of Gold NPs		
	(% + Standard Error)			(% + Standard Error)		
	Low Dose 1.35 μg/mL	Medium Dose 2.03 μg/mL	High Dose 2.7 μg/mL	Low Dose 1.35 μg/mL	Medium Dose 2.03 μg/mL	High Dose 2.7 μg/mL
***Staphylococcus aureus***	46.4 ± 0.4	33.75 ± 1.2	22.4 ± 0.8	53.1 ± 0.6	40 ± 1.3	23.7 ± 0.8
***Escherichia coli***	45.2 ± 1.4	26.6 ± 0.8	4.2 ± 0.9	47.6 ± 1.3	32.2 ± 0.8	6.2 ± 0.9
***Klebsiella pneumonia***	38.3 ± 0.2	22.8 ± 0.3	10.0 ± 0.2	42.7 ± 0.3	26.8 ± 0.1	13.8 ± 0.2
***Bacillus Subtilis***	57.8 ± 0.2	52.1 ± 0.1	45.2 ± 0.2	60.7 ± 0.2	56.3 ± 0.1	49.4 ± 0.2

**Table 2:** Calculation of zone of inhibition of (15 μL and 30 μL) of samples G1 and G2 of GNPs.

Micro Organisms	Organisms Category	Dose (μL)	Zone of Inhibition (mm)		
			Control	G2 NPs	G1 NPs
*Staphylococcus aureus*	Gram Positive	05	0	13	12
		15	0	20	23
		30	0	22	25
*Escherichia coli*	Gram Negative	05	0	10	11
		15	0	28	32
		30	0	31	35
